# Supplementary material for: Identification of Therapeutic Targets for Medulloblastoma by Tissue-Specific Genome-Scale Metabolic Model
Source: Molecules. 2023 Jan 12;28(2):779. doi: 10.3390/molecules28020779 (PMC9864031; doi:10.3390/molecules28020779)
Supplement: Supplementary file 1 [file molecules-28-00779-s001.zip › Supplementary Material 3.pdf]

Supplementary Material 3: Flux Sampling Results

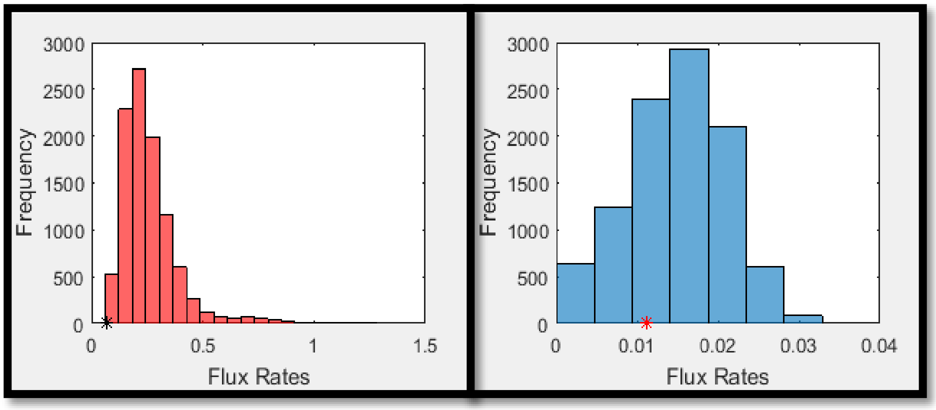

Figure S1. Lactate production (R11) in glycolysis for astrocyte. Flux sampling results for MB are shown in red while the results for healthy are shown in blue. Black asterisk on the red histogram indicates the flux result obtained using FBA for MB, the red asterisk on the blue histogram shows the flux rate for the healthy model.

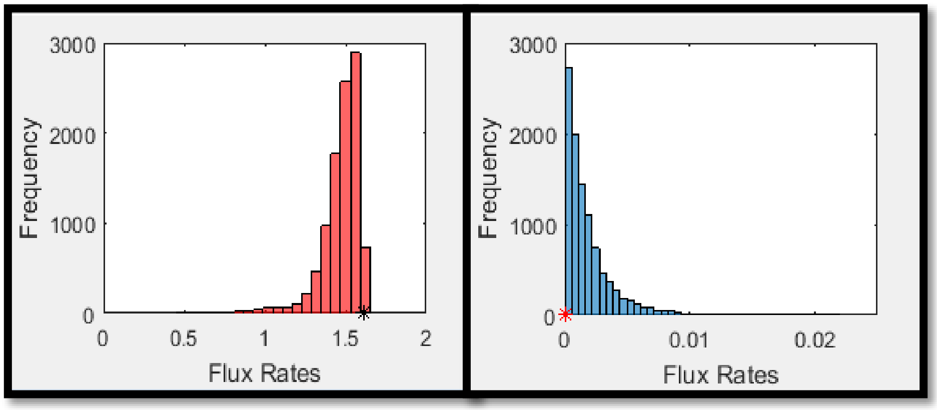

Figure S2. Lactate production (R56) in glycolysis for neuron. Flux sampling results for MB are shown in red while the results for healthy are shown in blue. Black asterisk on the red histogram indicates the flux result obtained using FBA for MB, the red asterisk on the blue histogram shows the flux rate for the healthy model.

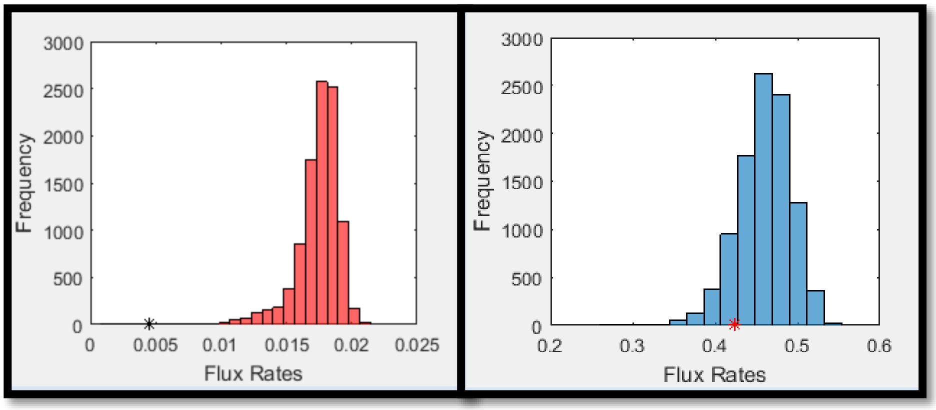

Figure S3. ATP production (R45) in OXPHOS for astrocyte. Flux sampling results for MB are shown in red while the results for healthy are shown in blue. Black asterisk on the red histogram indicates the flux result obtained using FBA for MB, the red asterisk on the blue histogram shows the flux rate for the healthy model.

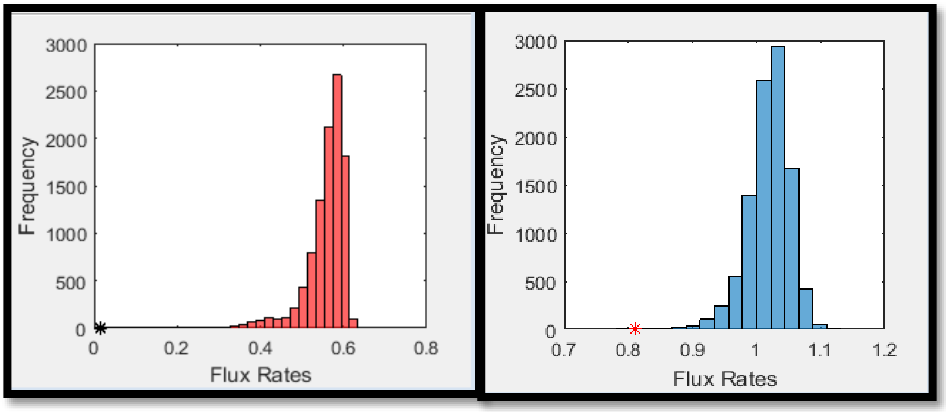

Figure S4. ATP production (R88) in OXPHOS for neuron. Flux sampling results for MB are shown in red while the results for healthy are shown in blue. Black asterisk on the red histogram indicates the flux result obtained using FBA for MB, the red asterisk on the blue histogram shows the flux rate for the healthy model.

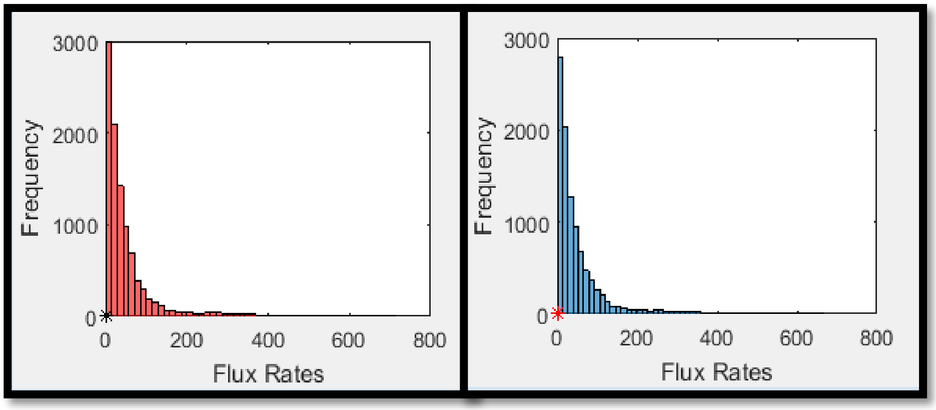

Figure S5. Citrate production (R25) in the TCA cycle for astrocyte. Flux sampling results for MB are shown in red while the results for healthy shown in blue. Black asterisk on the red histogram indicates the flux result obtained using FBA for MB, the red asterisk on the blue histogram shows the flux rate for the healthy model.

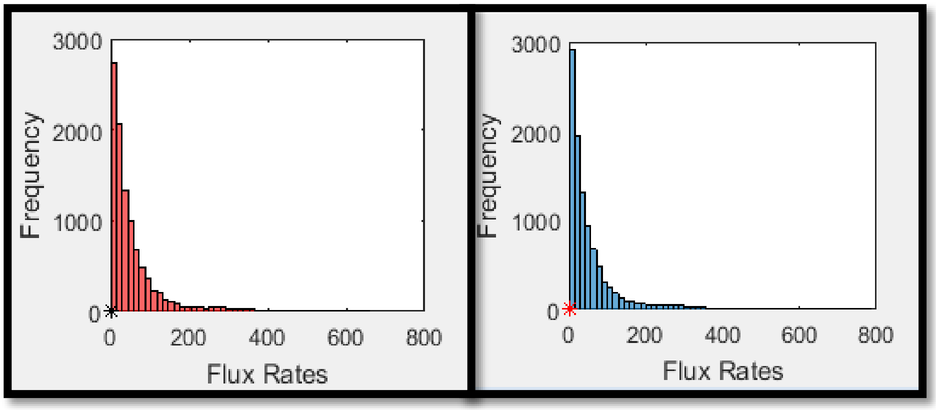

Figure S6. Citrate production (R69) in the TCA cycle for neuron. Flux sampling results for MB are shown in red. The results for healthy are shown in blue. Black asterisk on the red histogram indicates the flux result obtained using FBA for MB, the red asterisk on the blue histogram shows the flux rate for the healthy model.
